# Learning from MARQuIS: future direction of quality and safety in hospital care in the European Union

**DOI:** 10.1136/qshc.2008.029447

**Published:** 2009-01-26

**Authors:** O Groene, N Klazinga, K Walshe, C Cucic, C D Shaw, R Suñol

**Affiliations:** 1 Avedis Donabedian University Institute, Autonomous University of Barcelona, Spain, and CIBER Epidemiology and Public Health (CIBERESP); 2 Academic Medical Center, University of Amsterdam, Department of Social Medicine, Amsterdam, the Netherlands; 3 Herbert Simon Institute for Public Policy and Management, Manchester Business School, Manchester, UK; 4 The Dutch Institute for Health Care Improvement, the Netherlands; 5 European Society for Quality in Health Care, Limerick, Ireland

## Abstract

This article summarises the significant lessons to be drawn from, and the policy implications of, the findings of the Methods of Assessing Response to Quality Improvement Strategies (MARQuIS) project—a part of the suite of research projects intended to support policy established by the European Commission through its Sixth Framework Programme. The article first reviews the findings of MARQuIS and their implications for healthcare providers (and particularly for hospitals), and then addresses the broader policy implications for member states of the European Union (EU) and for the commission itself. Against the background of the European Commission’s Seventh Framework Programme, it then outlines a number of future areas for research to inform policy and practice in quality and safety in Europe. The article concludes that at this stage, a unique EU-wide quality improvement system for hospitals does not seem to be feasible or effective. Because of possible future community action in this field, attention should focus on the use of existing research on quality and safety strategies in healthcare, with the aim of combining soft measures to accelerate mutual learning. Concrete measures should be considered only in areas for which there is substantial evidence and effective implementation can be ensured.

For the past decade, European policy makers have been faced with a challenging contradiction. On the one hand, healthcare remains fundamentally a national responsibility of European Union (EU) member states, outside the competences and powers of the EU as set out in successive treaties. This position is strongly defended by national governments, which are understandably protective of their healthcare systems. Yet on the other hand, the rising tide of economic and social integration, driven both by EU policies such as the single market and by long-term societal trends in the family, employment, education, language and culture, have created increasing pressures for convergence, coherence and coordination in health system funding and provision. Since the European Commission first established its high-level reflection process on patient mobility and healthcare developments in 2003,^1^ and started the chain of events that culminated in the recent publication of a proposal for a directive on the application of patients’ rights in cross-border care,^2^ it has been increasingly clear that assuring the quality and safety of healthcare is a central concern for all stakeholders—the Commission itself, member states, health system funding agencies, healthcare providers, health professionals and patients. In commissioning a number of research projects to map, describe and analyse the quality of healthcare, and the policies and systems in place to assure quality, the Commission provided an essential evidence base for these policy developments, which has undoubtedly been influential in shaping the content of the current draft directive.^3^

This article summarises the significant lessons to be drawn from, and policy implications of, the findings of the Methods of Assessing Response to Quality Improvement Strategies (MARQuIS) project (box 1)—a part of the suite of research projects intended to support policy research established by the Commission through its Sixth Framework Programme. This article first explores the lessons for healthcare providers (and particularly for hospitals), and then turns to analysing the lessons and policy implications for EU member states and for the Commission itself. Finally, it outlines a number of future areas for research to inform policy and practice in healthcare quality and safety in Europe.

**Box 1: How the subject of this paper links to better patient care**

- The MARQuIS project is providing a comparative analysis on the impact of different quality management strategies in EU hospitals
- The results of the project are guiding the development of quality improvement strategies in order to improve patient care, and adding to the evidence base on the effectiveness of quality management systems

## KEY FINDINGS FROM THE MARQUIS PROJECT AND LESSONS FOR HOSPITALS

The MARQuIS project was designed to assess the value of different quality strategies, and to provide information on quality requirements for cross-border patients. In addition, it aimed to provide individual hospitals with information on the development of their quality strategies.

### Key findings of the study

Although the phenomena of cross-border care (which can be broadly grouped into five categories: temporary visitors abroad, people retiring as long-term residents to other countries, people living in border areas, people referred abroad for treatment, and people who seek treatment abroad themselves)^4^ has received considerable policy attention, our research confirmed that the overall phenomenon reflects only a small percentage of healthcare service delivery (less then 1% of total hospital admissions).^5^ In some regions and for some hospitals, however, the volume of cross-border care can be considerable, not only in terms of hospitalisations but also for emergency visits. For individual hospitals the issue of cross-border care may therefore be highly relevant. Moreover, the term cross-border patient itself can be confusing because a person working for a local company, but who is a citizen of another country, will not be considered a cross-border patient, although the expectations and needs of these patients may differ from those of local patients. Our research provided insight into the profile of cross-border patients, most of whom seek care for acute conditions or for emergency care. For hospitalised cross-border patients, the most frequent diagnoses involve the circulatory system or fractures. In addition, deliveries and other diagnoses related to pregnancy, pneumonia, appendicitis and other diseases of the digestive system are common diagnoses for this population.^5^

Cross-border patients’ needs are similar to those of other patients, but they have particular requirements that need to be fulfilled to ensure quality healthcare and safety for these patients.^6^ Information requirements with regard to the use of different languages are more pronounced among cross-border patients. Although hospitals use interpreters to improve communication with these patients, our study shows that in some cases it might be difficult for cross-border patients and professionals to communicate at the same level and share understanding during history taking, explanation of medications, and during discharge preparation. Moreover, informed consent procedures differ considerably between countries in terms of content and scope, which has practical implications (relating, for example, to participation in clinical trials) and raises potential legal issues related to the diversity of procedures. Differences can also be identified in medical procedures (such as the use of organs from non-heart patients, the scope of rehabilitation services and caesarean section on demand), and clinical practice guidelines may place emphasis on different aspects across countries. Cross-border patients may also be exposed to additional safety risks in view of prescription procedures for drugs that differ in name and dosage between countries, or in view of the difficulties of arranging medical transport between countries.

With regard to the quality strategies employed by the member states of the EU, the rate of progress varies considerably.^7^ ^8^ Although all countries implement the main quality strategies to some extent (such as accreditation systems, organisational quality management programmes, audit and internal assessment of clinical standards, patient safety systems, clinical practice guidelines, performance indicators and systems for getting patient views), three groups of countries can be identified. Countries where implementation is “well established” have been active in the implementation of a wide range of quality improvement strategies for years, and have well-established systems in place. “Recent adaptors” have recently established policies and strategies, and are in the process of consolidating their regulatory systems. “Slow starters” have begun initiatives in the field of quality improvement, but lack a coherent programme of government policy in this area. Member states belonging to the group of “recent adaptors” or “slow starters” are thus advised to study the existing experience of other member states with legislation in place, notably the impact of statutory legal requirements, on the implementation of quality improvement strategies in healthcare organisations. Member states with well-developed systems, on the other hand, could strengthen existing quality improvement legislation and other regulatory instruments to enhance and spread effective approaches to quality improvement.

Although research indicates some benefit of the regulatory approaches to quality improvement, this classification should not be confused with the implementation of quality improvement strategies in hospitals belonging to a given country. Analysis of the implementation of quality improvement strategies in European hospitals reveals that hospitals with a well-developed (and not so well-developed) quality improvement system can be identified in all countries.^9^ ^10^ The strategies studied in MARQuIS are effective at the hospital level, although the effect is nuanced depending on the level of analysis and the outputs under consideration. External pressure appears to be consistently associated with the implementation of quality improvement strategies at the hospital level. The quality improvement strategies under evaluation influence the implementation of different policies and procedures, and are, to varying extents, associated with the attainment of hospital output goals. Some of these associations, in particular with regard to patient safety and patient-centredness, however, may be confounded by hospital and country effects.^11^^–^13 Details of these findings are discussed in other articles in this supplement.

While most of these results are relevant for EU member states, some are also relevant for countries in other continents, for example in North America, Asia, and Australia. With regard to evaluating the effectiveness of quality improvement strategies, similar research projects in the USA and Australia deserve note,^14^ ^15^ and the exchange of methodologies and findings may accelerate research and quality improvement implementation in the different settings. It should also be noted that other regions provide cross-border care, for example between the USA and Canadian border,^16^ and that some healthcare providers in Asia market specialised healthcare services globally.^17^ In this context, the results of our research on cross-border care may also inform developments and quality improvement beyond the EU.

### Limitations of the study

There are a number of limitations of the study, which are discussed in detail in the different analyses presented elsewhere in this supplement. However, some general limitations will be addressed here. First, as in many other international studies on quality and safety in healthcare, it should be noted that there are substantial differences in the way member states organise their health services, and ensure quality and safety of care, and these differences pose substantial challenges to comparative research.^18^ Moreover, the use of different languages and terminology adds to this challenge. Although we made efforts to reduce these biases (for example, by using protocols for forward and backward translation in the design of the questionnaires), certain imperfections in comparative research on the organisation and impact of health services remain. Second, the countries participating in the project are not representative for all countries in the EU. Although efforts were made to select hospitals randomly, the low response rates in some countries limit the generalisability of the findings. Third, the main outputs of the MARQuIS study are based on a cross-sectional study design, since an experimental design would not have been appropriate in light of the research questions. The internal validity of the study is thus limited, and we cannot establish causality for the findings. Lastly, due to logistic and financial limitations we could not include clinical and patient-reported outcome measures in this study.

Nevertheless, it should be emphasised that theory and knowledge regarding the impact of quality improvement systems are at an early stage, and the MARQuIS project makes important contributions to further developing the evidence base in this field. Interestingly, researchers in Australia and the USA are using similar strategies to evaluate the effectiveness of their systems, to ensure the quality and safety of healthcare services.^19^ ^20^ In terms of assessing the external validity of the results and the applicability of the recommendations, it should be borne in mind that the findings are based on a substantial sample of European hospitals. In addition, to ensure clarity and feasibility of the full recommendations, a public consultation exercise was carried out involving project partners, country coordinators, members of the Advisory Council, the High Level Group, the Working Group on Patient Safety, researchers and selected European organisations. The results of the consultation exercise showed that overall, most recommendations were considered to be clear and feasible by the respondents. Individual recommendations that obtained lower ratings in terms of clarity or feasibility were revised. The full report on the consultation process is available on the MARQuIS website.^21^

### What can hospitals learn from MARQuIS to improve quality and safety?

The implementation of strategies and policies to improve healthcare quality and safety (such as systems for obtaining patients’ views, performance indicators, patient safety systems, clinical guidelines, accreditation schemes, audit or internal assessment of clinical standards, and organisational quality management programmes) may sometimes appear to be remote from clinical practice, but they do have an impact on the management of patients. Hospitals are thus encouraged to devote sufficient resources to quality improvement infrastructures, information systems and professional training in quality improvement. Although hospitals with a strong systematic approach to quality improvement are more likely to implement quality throughout the organisation, not all strategies in the quality toolbox are equally effective in all settings, and a combination of strategies appears to be more effective than a focus on a single strategy. Moreover, quality strategies require reinforcement at the clinical level to promote and adapt them to the specific context, such as the department, disease group or professional group.^22^

Structured guidelines may be developed or adapted within hospitals to systematically provide crucial information related to the administrative and clinical process for cross-border patients. Because many cross-border patients may not understand the language commonly used in a given setting, brochures on hospital organisation and (clinical) information leaflets could be translated into the language spoken by the most relevant groups of cross-border patients. In addition, hospitals may want to take stock of the languages spoken by their staff, and provide interpreters as necessary, in particular, to ensure that patients understand the information provided and can participate in decisions (for example, procedures for informed consent or shared clinical decision making). In order to ensure safety and continuity of care, prescriptions at discharge should use generic drug names, and should specify the dose of active component. Furthermore, at discharge, cross-border patients should receive enough medication to ensure continuity of treatment until the next point of care. Few hospitals appear to make sufficient use of patients’ knowledge. Most hospitals carry out periodic surveys on patients’ views, but the learning experience from these surveys is often not used to improve processes. Moreover, patients or patient groups are rarely involved in the development and evaluation of quality systems, although patients can make valuable contributions in this area. With regard to improving the quality and safety of cross-border care, hospitals should be aware that the needs of cross-border patients are similar to those of other patients, but that specific information and communication requirements exist and which should be promoted. The resource implications of these recommendations need to be assessed by individual hospitals in view of the volume of cross-border patients.

Although the emphasis on specific quality improvement strategies differs between countries and hospitals, the MARQuIS project, building on earlier EU-funded studies on quality of hospital care (COMAC and Biomed I and II), has again demonstrated the benefits of European research studies involving large samples of hospitals in an active way. Feedback from the hospitals on both the survey and the audit has been positive, and this type of action-oriented research not only has merits in producing new scientific knowledge, but also serves as an external “European” incentive for hospitals to reflect on their own experience, and compare themselves with colleagues. This seems especially true for concrete comparisons at the departmental level relating to specific clinical care processes. As such, an additional lesson to be learned is that similar European research projects should be more action based, thus enabling participating hospitals to benefit from the experience and enhance mutual learning.

## POLICY IMPLICATIONS FOR THE EU AND ITS MEMBER STATES

The MARQuIS project provides a wide range of evidence which should encourage both the European Commission and EU member states to adopt formal policies and strategies designed to assure and improve the quality of healthcare. In brief, at both the national and at organisational level, our findings suggest that these policies “work”—that is, they contribute to bringing about improvements in the quality of patient care. But beyond that, there are a number of more specific and focused lessons about both the direction of strategies or policies, and about their implementation, which deserve to be highlighted.

### Considerations at the level of EU member states

For EU member states, our findings do not suggest that there is a single approach or methodology for safety and quality measurement and improvement that should be adopted because of being demonstrably more effective than others, or because it is already predominant in use elsewhere. Rather, we found a multitude of different, overlapping and sometimes duplicative quality improvement initiatives, which often promoted, or focused on, different aspects or dimensions of quality and safety, and which were probably most effective when used in combination. However, the acceptance, or even promotion, of such multilateral and multifaceted strategies for quality improvement may mean that greater attention needs to be paid to their integration and internal consistency or coherence. For individual member states it is clear that there is much to be learned from the experiences of other member states in establishing healthcare quality improvement systems, and an opportunity to accelerate learning as well as progress significantly. It seems that efforts to provide a strong statutory framework for quality improvement should aim to embed quality improvement within existing health system funding and provision systems. This approach would provide resources to support the creation of improvement capacities in health systems and healthcare organisations, and would ensure that mechanisms exist to monitor implementation, follow up on compliance, and evaluate impact—all of which are necessary components of a strategy for healthcare quality improvement at the national level.

Strategies to improve quality and safety (such as systems for obtaining patients’ views, performance indicators, patient safety systems, clinical guidelines, accreditation schemes, audit or internal assessment of clinical standards, and organisational quality management programmes) promote different aspects of quality, and are most effective when used in combination. Member states in the process of developing their quality strategies are thus encouraged to study existing experiences, notably the impact of statutory legal requirements on the implementation of quality improvement strategies in healthcare organisations. In addition, the effect of external assessment programmes should be examined by countries that are developing their quality tools, given the effectiveness of such programmes. Further aspects that should be promoted are leadership, proper resource allocation, education and training, and planning and evaluation of quality improvement activities. Few hospitals currently provide opportunities for patient and public involvement in designing hospital services, and member states may want to address this with specific incentives.

**Box 2: Should the EU support the development of unique quality improvement systems, or instead help to strengthen existing systems?**

- EU member states have adapted a wide range of different strategies to safeguard the quality and safety of hospital services
- All these strategies appear to contribute to improving quality and safety; however, there is as much variation within, as there is between, countries in the extent to which hospitals have implemented such strategies
- Convergence of improvement systems should thus be promoted through the use of different strategies and requirements identified at the national level
- At this stage, a unique quality improvement system for EU member states may not be necessary or effective
- Nevertheless, further research should address how the systematic implementation of quality improvement strategies by hospitals in member states can be safeguarded
- Moreover, differences in the strategies applied should be made more transparent for citizens and patients

**Key messages**

- There are clear differences in the extent to which EU member states have formalised legislation and statutory requirements, and have implemented strategies to improve the quality of care. Some member states have substantial experience in this field, whereas others are just getting started. This does not mean, however, that hospitals in member states with a long history of legislation on quality improvement have systematically adopted this experience
- Hospitals with a well-developed quality improvement system can be found in all member states, and there is as much variation in the uptake of quality improvement strategies within countries as there is between countries
- Regarding the sovereignty of EU member states in the issues of healthcare legislation, at this stage a unique EU-wide quality improvement system does not seem to be feasible, nor are there guarantees that such a system would be effective. This is reflected in the current proposal for an EU directive on the application of patients’ rights in cross-border care, which affirms that member states should ensure the quality and safety of healthcare based on the “common values and principles in European Union health systems”
- Member states’ interpretation of these values and principles, however, may differ, and may lead to different rates of implementing quality and safety strategies. Moreover, given that it is impossible to know in advance which healthcare providers will supply services to cross-border patients in the future, these quality requirements should be extended to all healthcare facilities
- At the EU level, additional concrete measures may thus be introduced for specific quality requirements, as for example, in the field for radiology, blood and tissues, for which EU directives already exist
- At the same time, voluntary initiatives can be supported to assess and improve the quality and safety of cross-border care driven by institutions operating within specific cross-border agreements, or by those with a particular interest in attracting cross-border patients.
- Future action in this field should make use of the existing research on quality and safety strategies in healthcare, and should aim to combine “soft” measures to accelerate mutual learning, and consider concrete measures only in areas for which a substantial evidence base exists, and effective implementation is likely

In order to improve the quality and safety of cross-border care, two particular issues could be promoted by member states. First, professional criteria should determine eligibility for elective treatment abroad, and administrative criteria should evolve based on professional judgement. Second, member states should provide incentives for financiers and healthcare providers to collect data on long-term outcomes of cross-border services, even if these data need to be collected in different countries. Although not studied specifically in the MARQuIS project, EU member states may wish to assess the availability and requirements for quality improvement legislation beyond hospital services, for example, in areas such as primary healthcare, mental health, long-term care, and private healthcare provision.

### Policy implications at the EU level

**Summary points for further research**

- Support further research to develop and validate a classification model for quality improvement systems in hospitals
- Test associations of quality improvement systems and their impact on outcome assessed in terms of safety, clinical effectiveness and patients’ views
- Carry out a mapping exercise of existing organisational standards and criteria of European quality improvement systems
- Assess the implementation of current EU directives pertinent to hospital services
- Analyse and monitor volume and type of cross-border care in order to improve understanding of cross-border movement, its impact on quality, and organisational and professional cultures

The lessons and policy implications for the EU and the European Commission are perhaps more nuanced (box 2), and their adoption must clearly take account of the *Realpolitik* of the European institutions and stakeholders involved in shaping health policy—a necessity well demonstrated by the difficulties already encountered in framing the current draft directive on cross-border care. The research can be taken to confirm that greater coherence and convergence across and between member states in both the quality and safety of healthcare, and in the systems used to assure quality and safety, are at least desirable and might even be considered essential. However, coherence and convergence could be achieved either quickly through top-down directive action at the European level or slowly through bottom-up collaborative action between member states. The latter seems a more realistic prospect, and so attention is likely to turn to funding and promoting actions designed to support such evolutionary and voluntary convergence, at least in the short to medium term. However, as current initiatives in healthcare quality and safety cooperation develop further, it will become increasingly feasible at some point to contemplate the adoption of a more formal, directive-based mechanism for convergence in the future.

The EU may consider a number of steps to further advance healthcare quality and safety actions in Europe. In many member states, quality policies and strategies are developed by multiple stakeholders at both subnational and regional levels as well as at the state level. The EU may promote existing quality mechanisms initiated by governments, hospital management, healthcare financiers, professionals or patient and consumer organisations by enhancing synergies between the activities of various actors within and between countries. The EU may further facilitate exchange of information on the impact of existing external assessment programmes applied to hospitals. Further attention may focus on assessing full implementation in all EU member states of existing EU directives in the healthcare field. With regard to cross-border care, improvements at the EU level may be instigated by introducing consistent coding of cross-border patients, and by using clinical and administrative minimum data sets that include mandatory inter-country equivalent fields (such as country of origin, diagnosis and categories of cross-border care). The EU could further improve the cross-border patient’s experience by assessing the feasibility of specific actions, such as common content for informed consent in EU countries, or a standardised European (electronic) format for the discharge summary to improve communication between providers and patients.

## FUTURE DIRECTIONS FOR RESEARCH AND POLICY

The MARQuIS, Europe4Patients, and SYMPATIE projects have among them established that timely, applied research has an important part to play in informing and shaping EU policy on quality safety.^23^ Looking forward, it is encouraging that the European Commission’s Seventh Framework research programme contains a number of actions and initiatives grouped together as concerned with optimising the delivery of healthcare to European citizens. Topics include the implementation of research in clinical practice, patient safety in medication usage, continuity of care, chronic disease management, long-term care for the elderly, human resource planning, clinician working times and safety, and health outcome measurement.^24^ Taken together, this set of new research initiatives represents a substantial investment of research resources in the creation of new quality improvement techniques and methods, and will continue to build on the work already undertaken through the Sixth Framework Programme.

However, the collaborative, bottom-up approach to policy development which has been adopted may need to be matched by a more collaborative and networked approach to research, both by funding agencies and by researchers. Institutions such as the International Society for Quality in Health Care, the European Society for Quality in Health Care and the European Health Management Association, and national-level health research funding agencies, have a part in ensuring that the growing investment in quality and safety research at the national level is framed and used in ways that maximise international transferability and value. For example, in the UK, the National Institute for Health Research has just invested £10 million in founding two academic centres for research on patient safety and service quality, and is about to invest about £90 million in a series of academic centres for applied health research, much of whose work will address issues of quality in healthcare delivery. In the Netherlands over the past years, the government has invested up to €100 million in a series of national quality improvement and innovation programmes that involve a substantial number of providers in hospital care, home care, care for the elderly, nursing home care, mental health and public health services. In many other European countries, similar programmes or agencies exist or are being created, and opportunities for learning and exchange between them need to be sought and secured. In addition, in selected areas research and collaboration might be complemented by Community action.

At the EU health policy level a number of issues need to be considered to balance feasibility and effectiveness in the development of quality and safety strategies. The recent publication of the proposal for a European Directive on the application of patients’ rights in cross-border healthcare emphasises that when healthcare is provided, it is vital for patients to receive^2^

“clear information that enables patients to make informed choices about their health care, mechanisms for ensuring quality and safety of the health care that is provided, continuity of care between different treating professionals and organisations, and mechanisms to ensure appropriate remedies and compensation for harm arising from health care.”

The proposal, based as it is on the common values and principles in EU health systems,^25^ makes clear that the Directive does not aim to interfere with existing health and social security systems, nor to undermine the sovereignty of individual countries in issuing legislation on quality and safety issues in healthcare. Nevertheless, the fact that it is not possible to know in advance which healthcare provider will supply cross-border care blurs the distinction between quality improvement for cross-border patients, on the one hand, and general quality improvement efforts, on the other. Also, the interpretation of the common values and principles by different EU member states may differ and lead to different strategic choices as to how to ensure the quality and safety of healthcare.^26^ This may lead to the need for more European-wide cooperation in the field of quality improvement in the future, particularly in (but not restricted to) the issues identified in our research, namely, the implementation of effective quality and safety strategies to ensure that patients have access to key medical, financial, and practical information, and to ensure continuity of healthcare.

It is conceivable that in the future, additional concrete measures may be introduced for specific quality requirements, as is now the case in the fields of radiology, blood and tissues and cells, for which EU directives exist.^27^^–^29 The introduction of any concrete measures should be accompanied by supporting implementation and evaluation strategies. Looking beyond the policy debate, however, it should not be forgotten that ensuring the same level of quality and safety among all healthcare providers is desirable but unattainable, as demonstrated by persistent variations in medical practice and outcomes within member states and even within hospitals.^30^^–^32

## CONCLUSION

The MARQuIS study has produced the first assessment of the value of different quality strategies implemented across hospitals in EU member states. A key result is that member states and hospitals have implemented a range of different quality improvement strategies which appear to be effective in ensuring quality and safety. However, there is substantial variation within and between countries in the extent to which hospitals systematically implement these strategies.

In the context of cross-border care an important policy implication of the study is that, given the effectiveness of these strategies, it is not necessary at the EU level to develop a unique quality improvement system. Moreover, considering the variation in maturity classification of hospitals within countries, there is no reason to assume that strategy implementation at the EU level will be more effective than current approaches put forward by member states. On the other hand, the wide range of strategies applied, and the variability of their implementation, offer unique opportunities for further learning and exchange, which should be stimulated by the EU.

On the research side, the MARQuIS project provides new insight into the little-studied topic of cross-border care, and may help researchers to set the future research agenda. For quality improvement researchers, the development of a quality improvement classification system and the evaluation of its impact on patient-centredness, patient safety and hospital outputs offer new opportunities for comparative analyses of quality improvement strategies. In the future, this may lead to better knowledge of the effectiveness of quality improvement strategies, which may in turn inform hospital managers, purchasing agencies and regulators.
